# Treatment effects of low-frequency repetitive transcranial magnetic stimulation combined with motor relearning procedure on spasticity and limb motor function in stroke patients

**DOI:** 10.3389/fneur.2023.1213624

**Published:** 2023-08-11

**Authors:** Ruijun Chen, Yu Zhang, Xiaocheng Wang, Yunfei Zhao, Shasha Fan, Yanping Xue, Jing Zhao, Yinlian Liu, Pingzhi Wang

**Affiliations:** ^1^Department of Rehabilitation, Shanxi Bethune Hospital, Shanxi Academy of Medical Sciences, Tongji Shanxi Hospital, Third Hospital of Shanxi Medical University, Taiyuan, China; ^2^Department of Rehabilitation, Tongji Hospital, Tongji Medical College, Huazhong University of Science and Technology, Wuhan, China; ^3^Department of Traditional Chinese Medicine, Shanxi Bethune Hospital, Shanxi Academy of Medical Sciences, Tongji Shanxi Hospital, Third Hospital of Shanxi Medical University, Taiyuan, China; ^4^Department of Traditional Chinese Medicine, Tongji Hospital, Tongji Medical College, Huazhong University of Science and Technology, Wuhan, China; ^5^Department of Medical Record and Statistics, Shanxi Provincial People's Hospital, Taiyuan, China; ^6^College of Sports Rehabilitation, Shanxi Medical University, Jinzhong, China; ^7^Department of Medical Record Management, Shanxi Bethune Hospital, Shanxi Academy of Medical Sciences, Tongji Shanxi Hospital, Third Hospital of Shanxi Medical University, Taiyuan, China; ^8^Department of Medical Record Management, Tongji Hospital, Tongji Medical College, Huazhong University of Science and Technology, Wuhan, China

**Keywords:** stroke, repetitive transcranial magnetic stimulation, motor relearning procedure, modified Ashworth scale, Fugl-Meyer, motor evoked potential

## Abstract

**Objective:**

Limb paralysis, which is a sequela of stroke, limits patients' activities of daily living and lowers their quality of life. The purpose of this study was to investigate the effects of repetitive transcranial magnetic stimulation (rTMS) combined with a motor relearning procedure (MRP) on motor function and limb spasticity in stroke patients.

**Methods:**

Stroke patients were randomly divided into a combined treatment group (rTMS + MRP) and a control group (MRP) (*n* = 30 per group). The control group was given MRP in addition to conventional rehabilitation, and the combined treatment group was given 1 Hz rTMS combined with MRP. The treatment efficacy was assessed by the modified Ashworth scale (MAS), Fugl-Meyer motor function scale, and motor evoked potential (MEP) testing.

**Results:**

After 4 weeks of treatment, the Brunnstrom score, Fugl-Meyer lower extremity motor function, and Fugl-Meyer balance function were significantly higher in the combination treatment group compared to the control group, while the MAS score was lower in the combination treatment group compared to the control group. The MEP extraction rate was higher in the combined treatment group compared to the control group, while the threshold and central motor conduction time (CMCT) were lower in the combined treatment group compared to the control group.

**Conclusion:**

Low-frequency rTMS combined with MRP had better efficacy on spasticity and motor function in stroke patients with hemiparesis than MRP alone.

## Introduction

Stroke is one of the most common neurological diseases worldwide, especially in the elderly. Limb dysfunction is one of the main causes of disability in stroke, which seriously affects patients' activities of daily living (1). It has been reported that nearly 70.0% of stroke patients suffer from limb dysfunction (2). Lower limb motor function is a prerequisite for daily activities and is often impaired after stroke, resulting in limited functional activities (3, 4). Lower limb spasm is a common symptom of stroke (5) and a result of upper motor neuron syndrome (UMNS) (6). Approximately 30% of stroke patients experience spasms that require intervention (7), which, if not actively treated, may lead to high muscle tone, joint contracture, and abnormal movement patterns of the affected limb, resulting in impaired weight-bearing capacity of the limb, affecting the patient's balance and walking ability. Lower limb spasm and motor dysfunction seriously affect the quality of life of patients, and ways to effectively control lower limb spasm and improve lower limb motor function represent important issues that are actively discussed in rehabilitation medicine.

From minutes to months after brain injury, other brain regions may undergo changes due to afferent nerve block, release of inhibition, activity-dependent synapse changes, membrane excitability changes, and formation of new synaptic connections or exposure of existing connections (8, 9). The naturally occurring functional reorganization of the cerebral cortex is limited, and functional training is one of the most important factors to improve patients' functional recovery and enable them to adapt to the environment and live independently (10). To achieve functional reorganization, patients need to practice specific activities, and the more they practice, the easier the reorganization becomes. In the facilitation model, the hierarchical structure of the brain was used to explain abnormal movement patterns and spasms after brain injury. Various studies have confirmed that normal movement patterns can be included in rehabilitation therapy to promote the formation of normal motor functions. Motor relearning techniques, including drafting training, muscle strength training, and correct posture guidance training, can correct lower limb spasm in stroke patients.

Transcranial magnetic stimulation (TMS) is a non-invasive nerve modulation and stimulation technique that uses electromagnetic induction properties to modulate cerebral cortical excitability for short or long periods by applying locally time-varying magnetic pulses. TMS includes three stimulus paradigms: single-pulse TMS (sTMS), double-pulse TMS (pTMS), and repetitive TMS (rTMS). sTMS and pTMS are mainly used as research tools to locate cortical function and measure cortical excitability. rTMS is a sequence of TMS pulses delivered at the same intensity to a single cortical region, which is used to regulate brain activity and can induce long-term effects (facilitation or inhibition). Motor relearning procedure (MRP) regards the recovery of motor function after stroke as a process of relearning or retraining. Through visual, auditory, and tactile feedback, the normal motor mode is constantly strengthened and the abnormal motor mode is corrected, so the patients can constantly cooperate with the treatment to promote functional recovery.

The current rehabilitation methods for post-stroke spasm and limb movement disorders include rTMS and MRP (11, 12). rTMS can influence the function and behavior of the cortex by safely modifying neuronal activity in certain brain areas (13). TMS uses Faraday's electromagnetic induction principle to generate an induced current in the brain. Faraday discovered that a pulsed current through a coil creates a magnetic field and that the rate of change of this magnetic field determines the secondary current induced in the nearby conductor. Regarding TMS, the electrical pulse passing through the conducting coil increases to peak intensity for a short time (<1 ms) before decreasing back to zero. The transient current creates a magnetic field perpendicular to the plane of the coil that increases as fast as the current (up to ~2.5 T) and then drops rapidly. This transient magnetic field passes unhindered through the subject's scalp and skull, creating an induced current in the brain that is parallel to the plane of the stimulation coil but in the opposite direction of the coil current (14).

To create an electric field in the brain using electromagnetic induction, rTMS delivers a brief, strong pulsed current to a coil placed on the subject's head. The generated electric field then modulates neuron transmembrane potential, hence modulating neural activity (15, 16). Studies have shown that rTMS may be effective in treating lower limb spasms and in regulating cortical excitability in the leg motor region (17). MRP is mainly applied in rehabilitation therapy for the recovery of motor function after stroke in adults. This method considers the restoration of motor function after a stroke as a process of retraining or relearning. Through constant reinforcement of appropriate movement patterns and correction of incorrect movement patterns, as well as collaboration with therapy, MRP may help patients achieve functional recovery (18).

The main objectives of this research are (1) to investigate the improvement of lower limb spasm, motor function, and cortical function of patients with prolonged treatment of stroke with rTMS combined with MRP, and (2) to further determine whether combined rTMS and MRP therapy is more effective than MRP alone on lower limb spasm, motor function, and cortical excitability in stroke patients.

## Materials and methods

### Study population

In total, 60 stroke patients with hemiplegia who were hospitalized at Shanxi Bethune Hospital from November 2020 to November 2021 were selected. The inclusion criteria were as follows: (1) patients satisfied the diagnostic standards established by the Fourth National Academic Conference on Cerebrovascular Diseases, with confirmation by head CT and/or MRI (19); (2) first onset, unilateral hemiplegia, disease course of 1–6 months; (3) age: 30–80 years; (4) stable vital signs and no signs of disease progression; (5) able to follow instructions, with no obvious cognitive impairment; (6) the Brunnstrom stage of the damaged lower limb between II and V; and (7) according to the improved Ashworth spasm assessment, the lower limb muscle of the patient was in spasm with spasmodic foot ptosis varus gait (limited ankle dorsiflexion, with varus) and the grade reached level 2 or greater.

The exclusion criteria were as follows: (1) subarachnoid hemorrhage and transient ischemic attack; (2) severe cognitive dysfunction or communication dysfunction such that the patient was unable to cooperate; (3) patients with cochlear implants or pacemakers or who underwent metal implants, such as internal pulse generators; (4) epilepsy or family history of epilepsy; (5) patients who had received Type A botulinum toxin injection and/or take oral antispasmodic drugs simultaneously for the treatment of lower limb spasms; (6) patients with tetraplegia or bilateral paralysis, ankle joint contracture, or deep muscle atrophy; and (7) patients who developed new lesions or symptom exacerbations during the treatment.

A total of 60 stroke patients with hemiplegia who met the inclusion criteria were divided into an rTMS combined with an MRP treatment group (experimental group) and an MRP treatment group (control group) according to the random number table method, with each group consisting of 30 patients. Based on conventional rehabilitation treatment, the experimental group was treated with rTMS therapy combined with exercise-relearning technology for 4 weeks of rehabilitation treatment, and the control group was treated with exercise-relearning technology only.

### Treatment methods

#### Routine rehabilitation

Routine rehabilitation included physical factor therapy, neuro-promoting techniques (e.g., Bobath method, Brunnstrom technique, PNF technique, and Rood technique), bracing, exercise therapy, and other methods. The treatment lasted for 4 weeks, with 40 min of training once a day, 6 days a week.

#### MRP treatment

According to the conditions of the patients, the rehabilitation personnel conducted step-by-step and repeated training for the motor dysfunction and the disability of daily living ability, so the enrolled patients could establish muscle memory, and the patients were trained through oral, visual, and other instructions. Under the guidance of the rehabilitation teacher, the patients were trained on the following for 20–30 min every day for 4 weeks: (1) upper and lower limb function training involved holding objects, lower limb support training, shoulder movement control, lower limb training with acceptable weight, and ankle and knee joint plantar flexion training; (2) sitting, standing balance training, and walking training; and (3) pelvic function training, including pelvic horizontal lateral movement training, forward and backward tilt training, body rotation training, and pelvic control training.

#### rTMS treatment

A circular coil and a YRD CCY-I magnetic stimulation instrument (Ired Medical Equipment New Technology Co., Ltd. Wuhan, China) were used to deliver rTMS to the contralesional M1 of the abductor pollicis brevis. The stimulation site was the primary motor cortex region (M1 region) of the non-involved side of the brain. The location of M1 was placed according to the 10–20 international electroencephalography system. The location of M1 was the CZ at the intersection of the naso-occipitalline and temporo-parietal lines and that of M1 was 2 cm away from the CZ and 2 cm forward, with alternating left and right stimulation. The center point of the circular coil was directed to the M1 region of the left or right cortex of the patient, and a single stimulation was given. The peak intensity of the pulsed magnetic field was 3T, the stimulation frequency was l Hz, the intensity was 90% RMT, and 1,200 pulses were used. The stimulation time was 20 min once a day, 6 days a week for 4 weeks.

### Evaluation indicators

#### Muscle tone assessment

Limb muscle tone was assessed according to the modified Ashworth Spasm Scale (MAS). The MAS is the most widely used clinical scale to evaluate muscle tone, which is used by countries to evaluate muscle tone, the effectiveness of rehabilitation interventions, and to guide rehabilitation and other treatments (20). Grades 0, I, I^+^, II, III, and IV were scored as 0, 1, 2, 3, 4, and 5, respectively. Muscle tension that was restored or reduced by more than two points was defined as a significant effect; a muscle tone score that was reduced by one point was defined as effective; and no reduction in muscle tone was defined as invalid.

#### Evaluation of lower limb motor function

The Fugl-Meyer Assessment Scale (FMA) was used for the assessment of lower limb function. Designed by Fugl-Meyer et al. (21), the FMA is a global assessment measure used to quantitatively assess limb recovery in hemiplegia after stroke. The quantitative rating scale designed based on the Brunnstrom rating scale is currently the most widely accepted and widely used evaluation method. The assessment scale has 17 items, with a total possible score of 34 points; each item has 3 points (0–2), with higher scores indicating better motor function, and lower scores indicating weaker motor function of the lower limbs.

#### Motor evoked potential detection

rTMS had been conducted using a standard protocol (22). The target muscles were slightly activated voluntarily during the cortical stimulation. Foraminal electromagnetic stimulation was conducted using the same stimulator to obtain the peripheral motor conduction time. Following the stimulation of the motor cortex (area M1), rTMS can cause muscle contraction in the affected limb, which allows clear motor-evoked potential (TMS-MEP), motor threshold (MT), and central motor conduction time (CMCT) variables to be recorded. Relevant data can be obtained by analyzing the measured signals and used to examine the corticospinal and cortical ball motor pathways to investigate functional integrity in diverse neurological disorders (23). The MT was calculated as the minimum stimulator output required to elicit a motor-evoked potential (MEP) >50 μV peak-to-peak amplitude in at least 5 of 10 consecutive trials. Latency was defined as the time from the onset of stimulation to the onset of CMAP (mixed muscle action potential). Then, CMCT was calculated by subtracting the peripheral motor conduction time, which was defined as the shortest latency achieved by the foraminal stimulation from the shortest cortical latency obtained by the cortical stimulation. MEPs generated by rTMS can be used to objectively evaluate the motor function of stroke patients (24).

Rehabilitation assessment, scale measurement, and MEP detection were performed before treatment and at 2 and 4 weeks after treatment by an investigator who was blinded to the patient grouping.

### Statistical analysis

The SPSS 23.0 software package (IBM Corp.) was used for statistical analysis. Quantitative data consistent with a normal distribution are presented as the mean ± SD, while those inconsistent with a normal distribution are presented as the median (inter-quartile range). The ratios of constituents were used to express qualitative data. The *t-*test was used if the comparison of quantitative data between the two groups was compatible with the homogeneity of normality and variance; otherwise, the rank sum test was used. The χ^2^ test was used to compare the qualitative data between the two groups. Comparison between the two groups before and after the intervention was conducted *via* repeated measurement ANOVA. The *p*-values of <0.05 indicated a statistically significant difference.

## Results

### Patient characteristics

The clinical characteristics of the patients are summarized in Table 1. In total, 30 patients were randomly assigned to either the experimental group (rTMS + MRP) or the control group (MRP), both of which consisted of patients with subacute and convalescent hemiplegia due to stroke. The two groups were not significantly different in terms of sex, age, educational level, stroke subtype, location of brain lesion, time from onset to treatment, paretic side, and severity of limb hemiparesis (all *P* > 0.05), as shown in Table 1.

**Table 1:** Characteristics of participants.

**Variables**	**Control group (*n* = 30)**	**Experimental group (*n* = 30)**	***P*-value**
Gender			0.787
Male	19	20	
Female	11	10	
Age (years), mean ± standard deviation	58.83 ± 8.60	58.13 ± 10.75	0.782
Education level			0.624
Primary school	11	15	
Junior high school	9	5	
Senior high school	6	6	
Undergraduate	4	4	
Stroke subtype			0.606
Cerebral hemorrhage	16	14	
Cerebral infarction	14	16	
Location of brain lesion			0.436
Left brain	18	15	
Right brain	12	15	
Time from the onset to treatment (months), median [inter-quartile range]	20.5 [13.0–31.0]	22.0 [17.0–40.0]	0.222
Paretic side			0.598
Right	11	13	
Left	19	17	
The severity of limb hemiparesis			0.796
Moderate (BRS II-III)	14	15	
Severe (BRS I)	16	15	

### Muscle tension assessment

**Figure 1:**
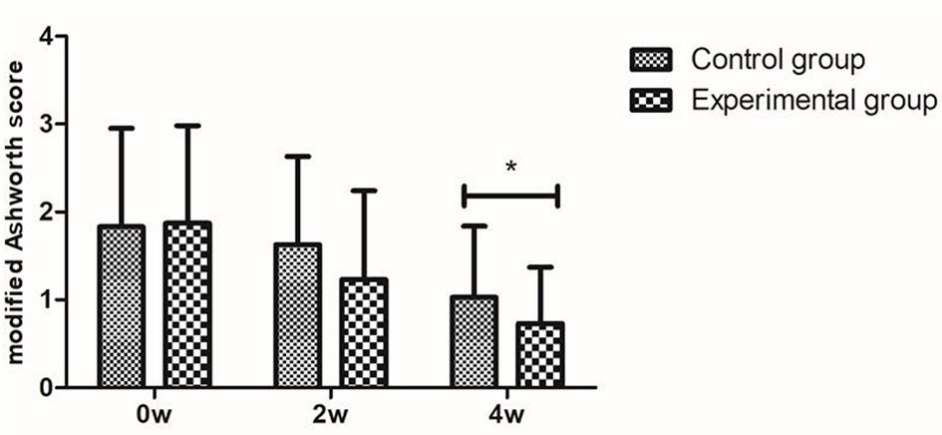
Muscle tone assessment: modified Ashworth score. *P < 0.05 with groups.

The muscular tension of both the control and experimental groups gradually increased. Additionally, there was no distinction between the two groups in terms of any index before the therapy (*P* > 0.05). The modified Ashworth score of the experimental group (1.03 ± 0.81) was lower than that of the control group (0.73 ± 0.64) following 4 weeks of therapy (*P* < 0.05) (Figure 1).

### Assessment of lower limb motor function

**Figure 2:**
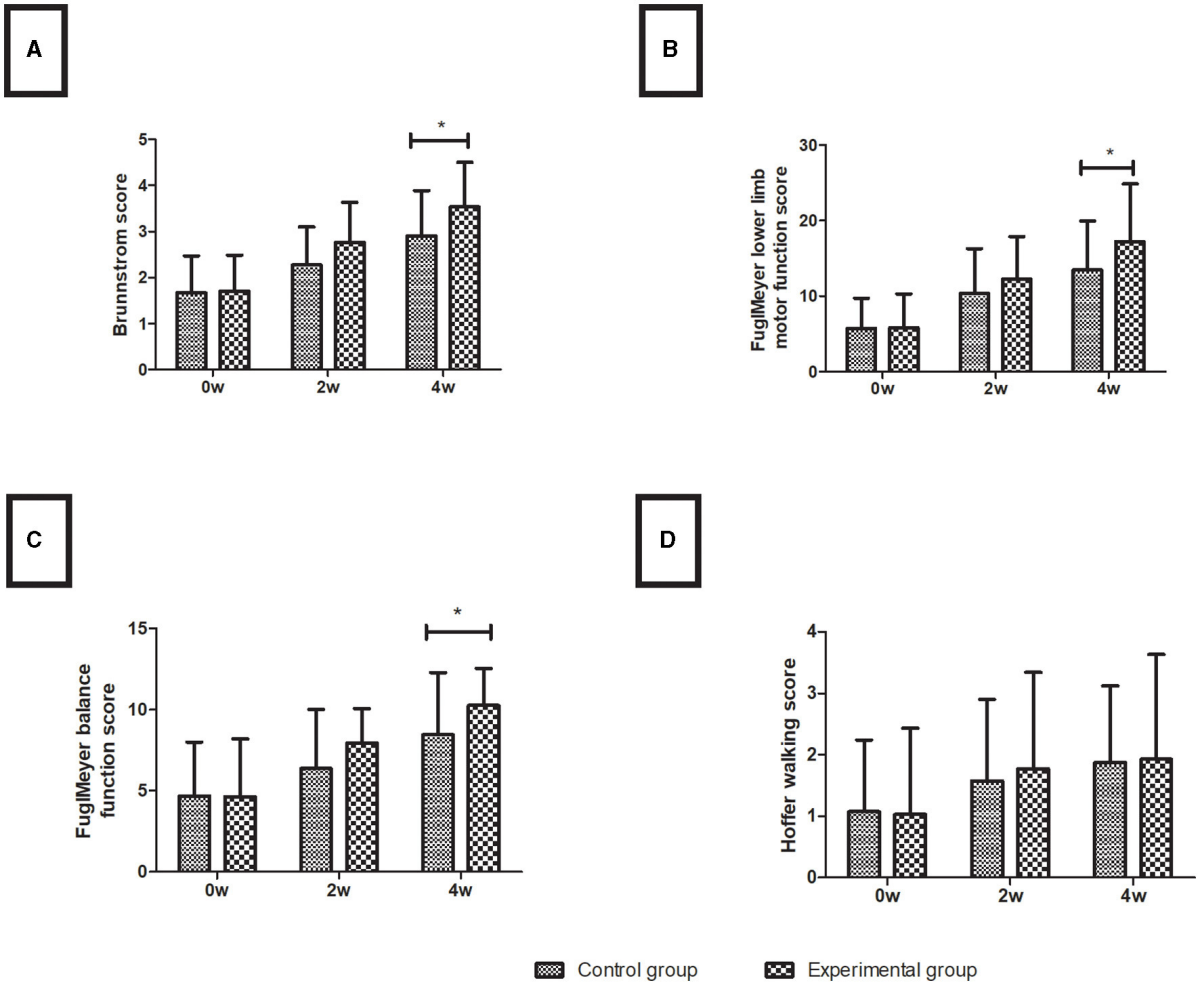
Lower limb motor function assessment. **(A)** Brunnstrom score; **(B)** Fugl-Meyer lower limb motor function score; **(C)** Fugl-Meyer balance function score; **(D)** Hoffer walking score. Data are presented in the form of mean ± SD. *P < 0.05 with groups.

In both the control and experimental groups, all measures of lower limb motor function improved with time. Before therapy, the two groups did not differ significantly in any index (*P* > 0.05). After 4 weeks of treatment, the Brunnstrom score (3.53 ± 0.97), Fugl-Meyer lower limb motor function (17.23 ± 7.65), and Fugl-Meyer balance function (10.27 ± 2.27) of the experimental group were higher than those of the control group (2.90 ± 0.99, 13.47 ± 6.50, 8.47 ± 3.82, respectively) (all *P* < 0.05), and the Hoffer walking score was not different between the experimental group (1.93 ± 1.70) and the control group (1.87 ± 1.25) in the motor function assessments (*P* > 0.05), shown in Figure 2.

### Motor evoked potential detection

**Figure 3:**
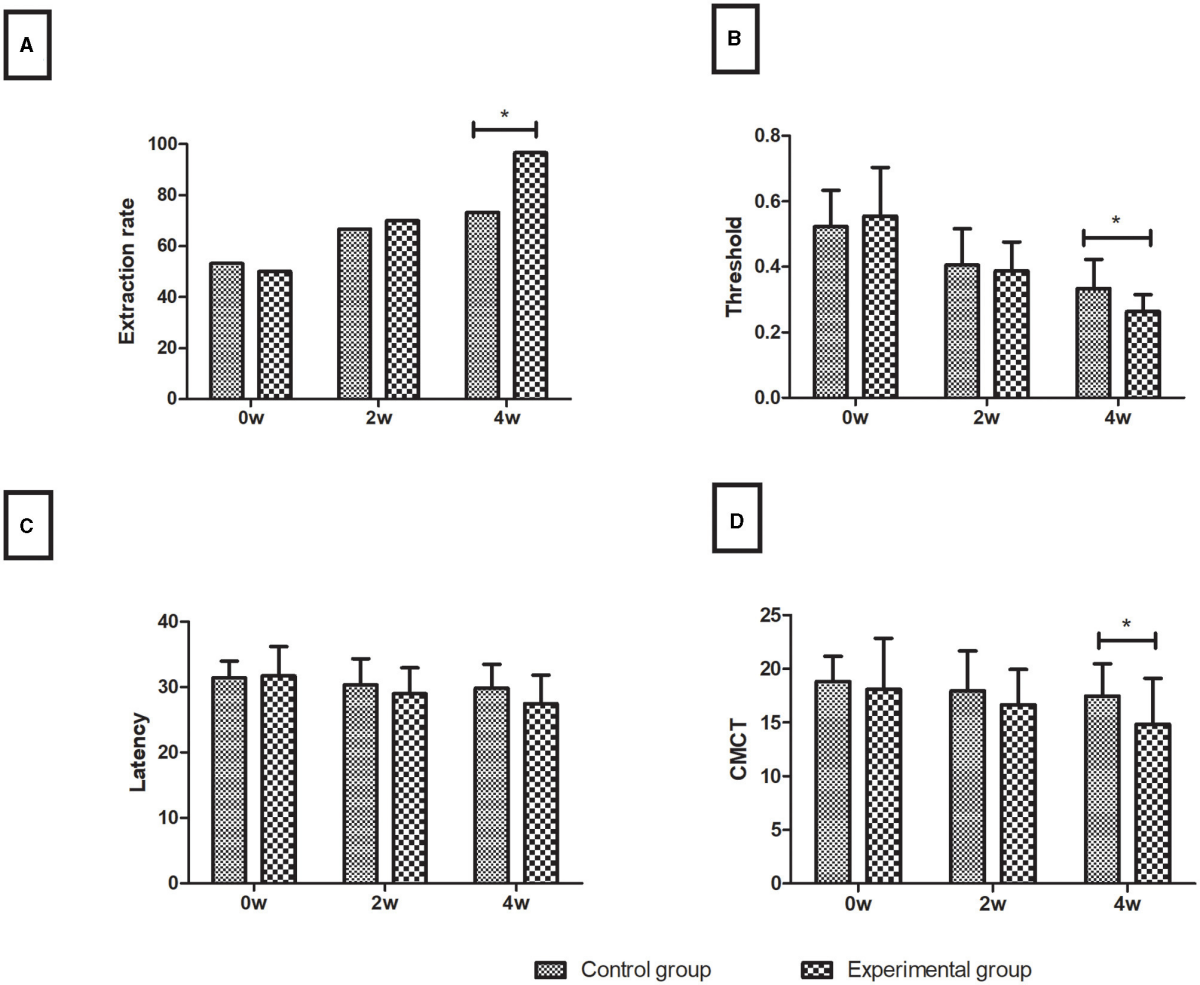
Motor evoked potentials. **(A)** Extraction rate (%); **(B)** Threshold; **(C)** Latency; **(D)** CMCT. Data are presented in the form of mean ± SD. *P < 0.05 with groups.

Over time, the magnetic stimulation motor-evoked potential (TMS-MEP) indicators were improved to varying degrees in both the control and experimental groups. The two groups showed no significant difference in any index before treatment (*P* > 0.05). After 4 weeks of treatment, the extraction rate of the experimental group (96.7%) was higher than that of the control group (73.3%), the threshold (0.26 ± 0.05) and CMCT (14.84 ± 4.30) of the experimental group were lower than those of the control group (0.33 ± 0.09 and 17.46 ± 3.03, respectively) (all *P* < 0.05), and the latency was insignificant between the experimental group (27.45 ± 4.35) and the control group (29.81 ± 3.68) in the assessment of limb functions (*P* > 0.05), as shown in Figure 3.

## Discussion

rTMS has been reported to treat muscle spasticity in stroke patients (24–29). The purpose of our study was to explore whether the efficacy of rTMS combined with MRP was better than that of MRP alone in 60 stroke patients with hemiplegia. To evaluate patient recovery, it is necessary to investigate whether the motor function of lower limbs can be restored (19). MRP treatment significantly improves motor function and alleviates spastic disorders in stroke patients. Although traditional rehabilitation therapy can relieve the symptoms of limb spasm to a certain extent, it cannot improve the excitability of the cerebral cortex on the affected side of the patient, and the therapeutic effect is limited. In our study, low-frequency rTMS was used to inhibit the M1 region of the non-involved side. After 4 weeks of treatment, the rTMS combined with MRP can increase cerebral cortex excitability on the affected side or decrease it on the healthy side, effectively enhancing the motor function (e.g., Fugl-Meyer lower limb motor function and Fugl-Meyer balance function) and muscle spasticity (lower modified Ashworth score) of affected limbs in stroke patients and show a more obvious effect (higher extraction rate and lower threshold and CMCT) than exercise relearning therapy alone.

According to the principle of hemispheric competitive inhibition (25, 30, 31), hemispheric cortical excitability in the afflicted area decreases after stroke, and the healthy hemisphere presents an increase in cortical excitability due to the transcallosal inhibition loss of the affected hemisphere, thus aggravating the inhibition of the affected hemisphere by the healthy hemisphere. Therefore, improving cerebral cortex excitability on the affected side or decreasing it on the healthy side can promote the recovery of limb function after stroke (32, 33). Our study found that 1 Hz rTMS treatment on the healthy side of the cerebral hemisphere can significantly improve limb spasm after stroke and promote limb functional rehabilitation, which may be related to the fact that low-frequency rTMS can inhibit the excitability of the M1 region on the healthy side, reduce its inhibitory effect on the affected cortex, and indirectly enhance the excitability of the M1 cortex on the affected side (29, 34, 35). In other words, after inhibiting the non-affected side, the affected side's velocity-dependent stretch reflex was enhanced, tendon hyperreflexia was relieved, the balance of bilateral cerebral hemisphere functions was restored, and muscle tension was reduced. Meanwhile, the cortical excitability cerebral hemisphere of the affected person was further improved (36). Lin et al. (24) found that rTMS can effectively improve the motor function of the impacted limb in stroke patients. rTMS can stimulate the excitability of the cerebral cortex and promote synaptic activity and remodeling, thus accelerating the recovery of cognitive impairment and enhancing the motor function and spasticity of stroke patients (37). Rastgoo et al. (27) found that low-frequency rTMS over the M1 of unaffected lower limbs can reduce lower limb spasticity and improve motor dysfunction caused by lower limb spasticity in stroke patients. This result is consistent with the findings of Li et al. (26), which showed that low-frequency rTMS stimulation of the contralateral side decreased the MAS score and increased the FMA score in stroke patients after therapy, suggesting that low-frequency rTMS reduced patients' muscle spasticity and limb dyskinesia.

This study has several limitations. First, we did not conduct long-term observation and follow-up on the subjects; therefore, long-term efficacy needs to be observed in further studies. Second, the number of cases included in this study was small. Third, due to equipment configuration, the YRD CCY-I magnetic stimulation instrument uses a circular coil instead of the commonly used figure-eight transcranial magnetic stimulation coil.

In conclusion, rTMS combined with MRP showed better efficacy than MRP in treating muscle spasticity and limb dyskinesia in stroke patients. The combination of rTMS and MRP may be a beneficial adjunctive technique for motor neurorehabilitation in stroke patients with muscle spasticity and limb dyskinesia.

## Data availability statement

The raw data supporting the conclusions of this article will be made available by the authors, without undue reservation.

## Ethics statement

The studies involving human participants were reviewed and approved by Shanxi Bethune Hospital, Shanxi Academy of Medical Sciences, Tongji Shanxi Hospital, Third Hospital of Shanxi Medical University. The patients/participants provided their written informed consent to participate in this study.

## Author contributions

YL and PW: conceptualization. YZhang: methodology. XW: formal analysis. YZhao: investigation. SF: data curation. RC: writing—original draft preparation. YL: funding acquisition. RC, YL, and PW: writing—review and editing. YX and JZ: project administration. All authors have read and agreed to the published version of the article.

## References

[1] Dimyan MA Cohen LG. Neuroplasticity in the context of motor rehabilitation after stroke. Nat Rev Neurol. (2011) 7:76–85. 10.1038/nrneurol.2010.200 21243015 PMC4886719

[2] Yan R Zhang Y Lim J Yang F Zhou L Lyu D. The effect and biomechanical mechanisms of intradermal needle for post-stroke hemiplegia recovery: study protocol for a randomized controlled pilot trial. Medicine. (2018) 97:e0448. 10.1097/MD.0000000000010448 29668611 PMC5916658

[3] Ji SG Kim MK. The effects of mirror therapy on the gait of subacute stroke patients: a randomized controlled trial. Clin Rehabil. (2015) 29:348–54. 10.1177/0269215514542356 25023068

[4] Li Y Wei Q Gou W He C. Effects of mirror therapy on walking ability, balance and lower limb motor recovery after stroke: a systematic review and meta-analysis of randomized controlled trials. Clin Rehabil. (2018) 32:1007–21. 10.1177/0269215518766642 29644880

[5] Singer JC Nishihara K Mochizuki G. Does poststroke lower-limb spasticity influence the recovery of standing balance control? a 2-year multilevel growth model. Neurorehabil Neural Repair. (2016) 30:626–34. 10.1177/1545968315613862 26507437

[6] Mayer NH. Clinicophysiologic concepts of spasticity and motor dysfunction in adults with an upper motoneuron lesion. Muscle Nerve Suppl. (1997) 6:S1–13. 9826979

[7] Sarikaya H Ferro J Arnold M. Stroke prevention–medical and lifestyle measures. Eur Neurol. (2015) 73:150–7. 10.1159/000367652 25573327

[8] Rothwell JC. Plasticity in the human motor system. Folia Phoniatr Logop. (2010) 62:153–7. 10.1159/000314030 20460927 PMC2998171

[9] Jones EG Pons TP. Thalamic and brainstem contributions to large-scale plasticity of primate somatosensory cortex. Science. (1998) 282:1121–5. 10.1126/science.282.5391.1121 9804550

[10] Kim Y Lai B Mehta T Thirumalai M Padalabalanarayanan S Rimmer JH. Exercise training guidelines for multiple sclerosis, stroke, and parkinson disease: rapid review and synthesis. Am J Phys Med Rehabil. (2019) 98:613–21. 10.1097/PHM.0000000000001174 30844920 PMC6586489

[11] Munari D Serina A Disarò J Modenese A Filippetti M Gandolfi M. Combined effects of backward treadmill training and botulinum toxin type A therapy on gait and balance in patients with chronic stroke: a pilot, single-blind, randomized controlled trial. NeuroRehabilitation. (2020) 46:519–28. 10.3233/NRE-203067 32508341

[12] Badr M Al-Otaibi S Alturki N Abir T. Detection of heart arrhythmia on electrocardiogram using artificial neural networks. Comput Intell Neurosci. (2022) 2022:1094830. 10.1155/2022/1094830 36035826 PMC9410968

[13] Yang F Chen L Wang H Zhang J Shen Y Qiu Y. Combined contralateral C7 to C7 and L5 to S1 cross nerve transfer for treating limb hemiplegia after stroke. Br J Neurosurg. (2021) 35:1–4. 10.1080/02688697.2021.1910764 33843383

[14] Kobayashi M Pascual-Leone A. Transcranial magnetic stimulation in neurology. Lancet Neurol. (2003) 2:145–56. 10.1016/S1474-4422(03)00321-1 12849236

[15] Lee SA Kim MK. Effect of low frequency repetitive transcranial magnetic stimulation on depression and cognition of patients with traumatic brain injury: a randomized controlled trial. Med Sci Monit. (2018) 24:8789–94. 10.12659/MSM.911385 30513530 PMC6289027

[16] Nardone R Sebastianelli L Versace V Brigo F Golaszewski S Manganotti P. Repetitive transcranial magnetic stimulation in traumatic brain injury: evidence from animal and human studies. Brain Res Bull. (2020) 159:44–52. 10.1016/j.brainresbull.2020.03.016 32251693

[17] Tassinari CA Cincotta M Zaccara G Michelucci R. Transcranial magnetic stimulation and epilepsy. Clin Neurophysiol. (2003) 114:777–98. 10.1016/S1388-2457(03)00004-X 12738425

[18] Mori F Koch G Foti C Bernardi G Centonze D. The use of repetitive transcranial magnetic stimulation (rTMS) for the treatment of spasticity. Prog Brain Res. (2009) 175:429–39. 10.1016/S0079-6123(09)17528-3 19660671

[19] Harwin WS Murgia A Stokes EK. Assessing the effectiveness of robot facilitated neurorehabilitation for relearning motor skills following a stroke. Med Biol Eng Comput. (2011) 49:1093–102. 10.1007/s11517-011-0799-y 21779903

[20] Meseguer-Henarejos AB Sánchez-Meca J López-Pina JA Carles-Hernández R. Inter- and intra-rater reliability of the modified ashworth scale: a systematic review and meta-analysis. Eur J Phys Rehabil Med. (2018) 54:576–90. 10.23736/S1973-9087.17.04796-7 28901119

[21] Fugl-Meyer AR Jääskö L Leyman I Olsson S Steglind S. The post-stroke hemiplegic patient. 1 a method for evaluation of physical performance. Scand J Rehabil Med. (1975) 7:13–31. 10.2340/1650197771331 1135616

[22] Groppa S Oliviero A Eisen A Quartarone A Cohen LG Mall V. practical guide to diagnostic transcranial magnetic stimulation: report of an IFCN committee. Clin Neurophysiol. (2012) 123:858–82. 10.1016/j.clinph.2012.01.010 22349304 PMC4890546

[23] Mazzoleni S Duret C Grosmaire AG Battini E. Combining upper limb robotic rehabilitation with other therapeutic approaches after stroke: current status, rationale, and challenges. Biomed Res Int. (2017) 2017:8905637. 10.1155/2017/8905637 29057269 PMC5615953

[24] Lin YN Hu CJ Chi JY Lin LF Yen TH Lin YK. Effects of repetitive transcranial magnetic stimulation of the unaffected hemisphere leg motor area in patients with subacute stroke and substantial leg impairment: a pilot study. J Rehabil Med. (2015) 47:305–10. 10.2340/16501977-1943 25679340

[25] Kondo T Kakuda W Yamada N Shimizu M Hagino H Abo M. Effect of low-frequency rTMS on motor neuron excitability after stroke. Acta Neurol Scand. (2013) 127:26–30. 10.1111/j.1600-0404.2012.01669.x 22494271

[26] Li D Cheng A Zhang Z Sun Y Liu Y. Effects of low-frequency repetitive transcranial magnetic stimulation combined with cerebellar continuous theta burst stimulation on spasticity and limb dyskinesia in patients with stroke. BMC Neurol. (2021) 21:369. 10.1186/s12883-021-02406-2 34560841 PMC8461848

[27] Rastgoo M Naghdi S Nakhostin Ansari N Olyaei G Jalaei S Forogh B. Effects of repetitive transcranial magnetic stimulation on lower extremity spasticity and motor function in stroke patients. Disabil Rehabil. (2016) 38:1918–26. 10.3109/09638288.2015.1107780 26878554

[28] Blesneag AV Slăvoacă DF Popa L Stan AD Jemna N Isai Moldovan F. Low-frequency rTMS in patients with subacute ischemic stroke: clinical evaluation of short and long-term outcomes and neurophysiological assessment of cortical excitability. J Med Life. (2015) 8:378–87. 26351545 PMC4556924

[29] Mansur CG Fregni F Boggio PS Riberto M Gallucci-Neto J Santos CM. sham stimulation-controlled trial of rTMS of the unaffected hemisphere in stroke patients. Neurology. (2005) 64:1802–4. 10.1212/01.WNL.0000161839.38079.92 15911819

[30] Mukherjee A Chakravarty A. Spasticity mechanisms - for the clinician. Front Neurol. (2010) 1:149. 10.3389/fneur.2010.00149 21206767 PMC3009478

[31] Li S. Spasticity, motor recovery, and neural plasticity after stroke. Front Neurol. (2017) 8:120. 10.3389/fneur.2017.00120 28421032 PMC5377239

[32] Sebastian R Saxena S Tsapkini K Faria AV Long C Wright A. Cerebellar tDCS: a novel approach to augment language treatment post-stroke. Front Hum Neurosci. (2016) 10:695. 10.3389/fnhum.2016.00695 28127284 PMC5226957

[33] Dimyan MA Cohen LG. Contribution of transcranial magnetic stimulation to the understanding of functional recovery mechanisms after stroke. Neurorehabil Neural Repair. (2010) 24:125–35. 10.1177/1545968309345270 19767591 PMC2945387

[34] Fisicaro F Lanza G Grasso AA Pennisi G Bella R Paulus W. Repetitive transcranial magnetic stimulation in stroke rehabilitation: review of the current evidence and pitfalls. Ther Adv Neurol Disord. (2019) 12:1756286419878317. 10.1177/1756286419878317 31598137 PMC6763938

[35] Takeuchi N Chuma T Matsuo Y Watanabe I Ikoma K. Repetitive transcranial magnetic stimulation of contralesional primary motor cortex improves hand function after stroke. Stroke. (2005) 36:2681–6. 10.1161/01.STR.0000189658.51972.34 16254224

[36] Rossini PM Rossi S. Transcranial magnetic stimulation: diagnostic, therapeutic, and research potential. Neurology. (2007) 68:484–8. 10.1212/01.wnl.0000250268.13789.b2 17296913

[37] Solomons CD Shanmugasundaram V. A review of transcranial electrical stimulation methods in stroke rehabilitation. Neurol India. (2019) 67:417–23. 10.4103/0028-3886.258057 31085852

